# Supplementary material for: Multiple CH/π Interactions Maintain the Binding of Aflatoxin B1 in the Active Cavity of Human Cytochrome P450 1A2
Source: Toxins (Basel). 2019 Mar 12;11(3):158. doi: 10.3390/toxins11030158 (PMC6468651; doi:10.3390/toxins11030158)
Supplement: Supplementary file 1 [file toxins-11-00158-s001.pdf]

Supplementary Materials: Multiple CH/π Interactions Maintain the Binding of Aflatoxin B_1 in Active Cavity of Human Cytochrome P450 1A2

Jun Wu †, Sisi Zhu †, Yunbo Wu, Tianqing Jiang, Lingling Wang, Jun Jiang, Jikai Wen and Yiqun Deng *

Table S1. The docking score of CYP1A2 and the mutants.

Samples	GB/VI Score (kcal/mol)
WT	−8.98
T124A	−8.37
F125A	−8.58
F226A	−8.09
F260A	−8.81

Table S2. Contents of secondary structure elements of CYP1A2 and its mutants. CD spectra were analyzed by CONTINLL [1].

Samples	α-Helix	β-Sheet	Turns	Unordered
		%		
WT	51	9.1	16.1	23.8
T124A	60.4	5.8	14.4	19.4
F226A	61.1	5.8	15.2	17.9
F260A	47.8	11.3	17	23.9

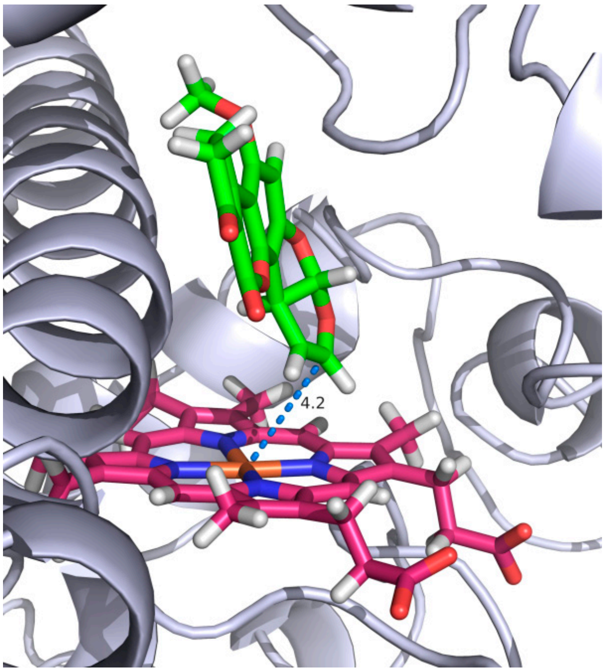

Figure S1. The side view of AFB1 conformation in the substrate pocket of CYP1A2.

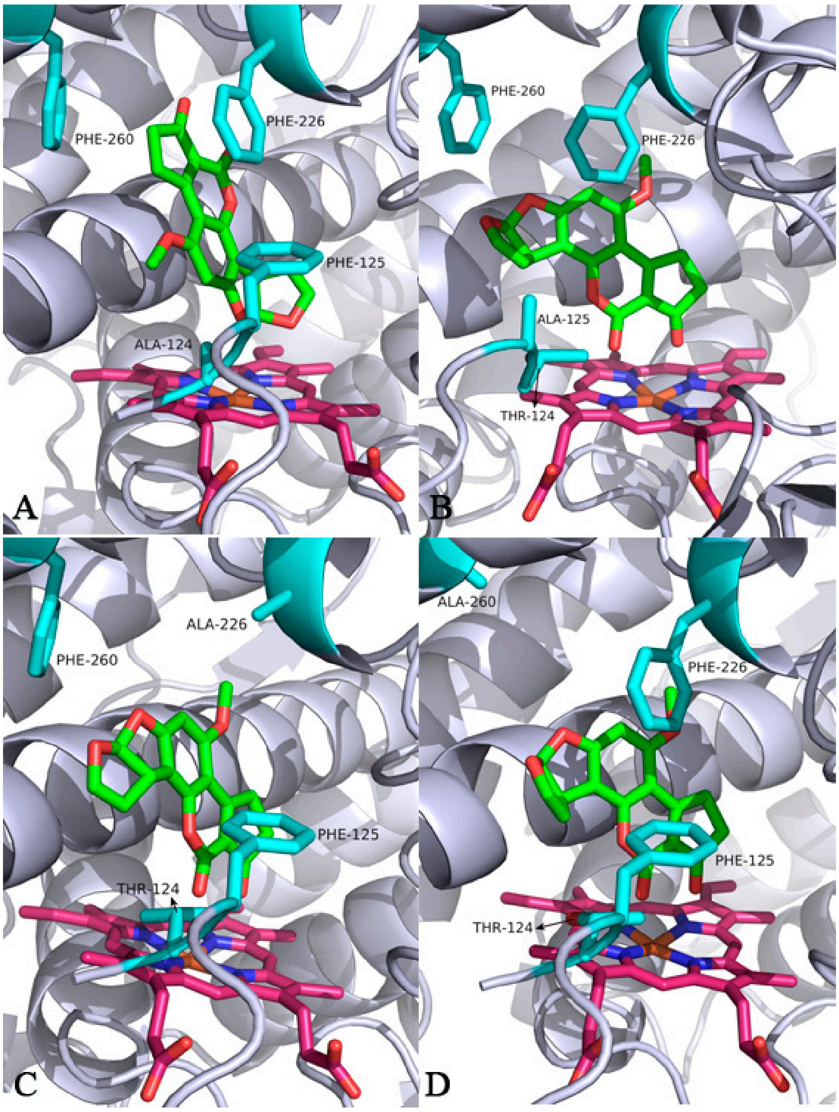

Figure S2. The docking models of AFB1 into CYP1A2 mutants. The docking conformation of AFB1 in T124A (**A**), F125A (**B**), F226A (**C**), and F260A (**D**).

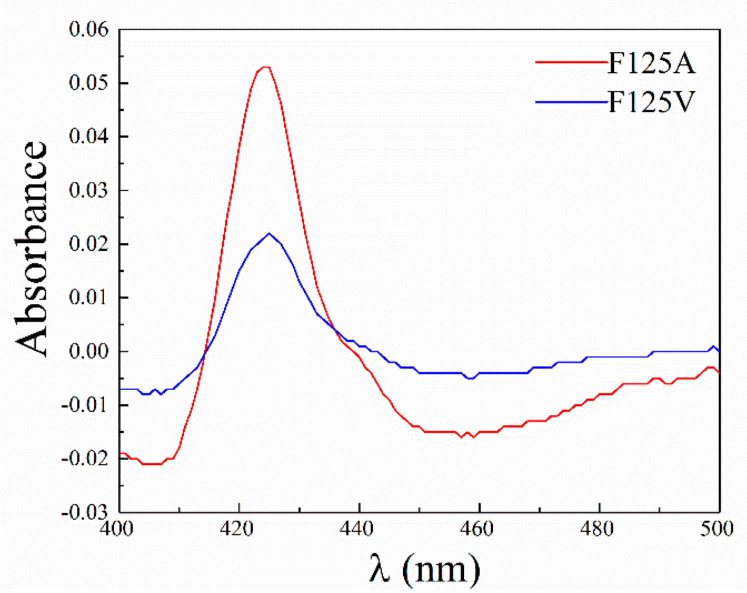

Figure S3. The Fe^{2+}·CO vs. Fe^{2+} difference spectra of F125A and F125V.

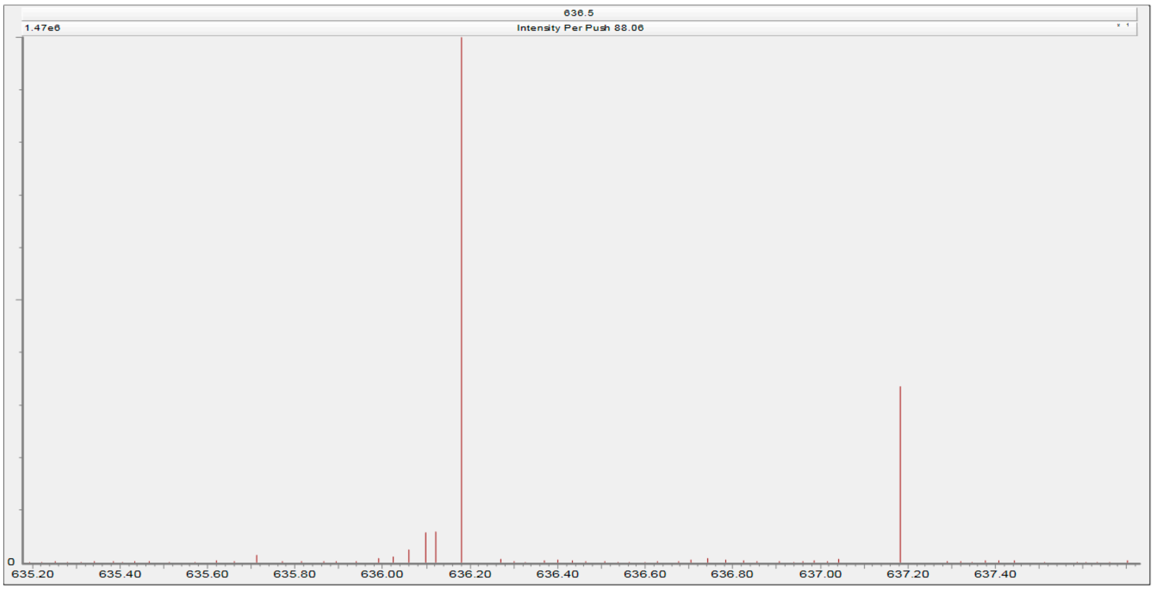

Figure S4. The parent ion of AFBO-GSH with *m*/*z* 636.

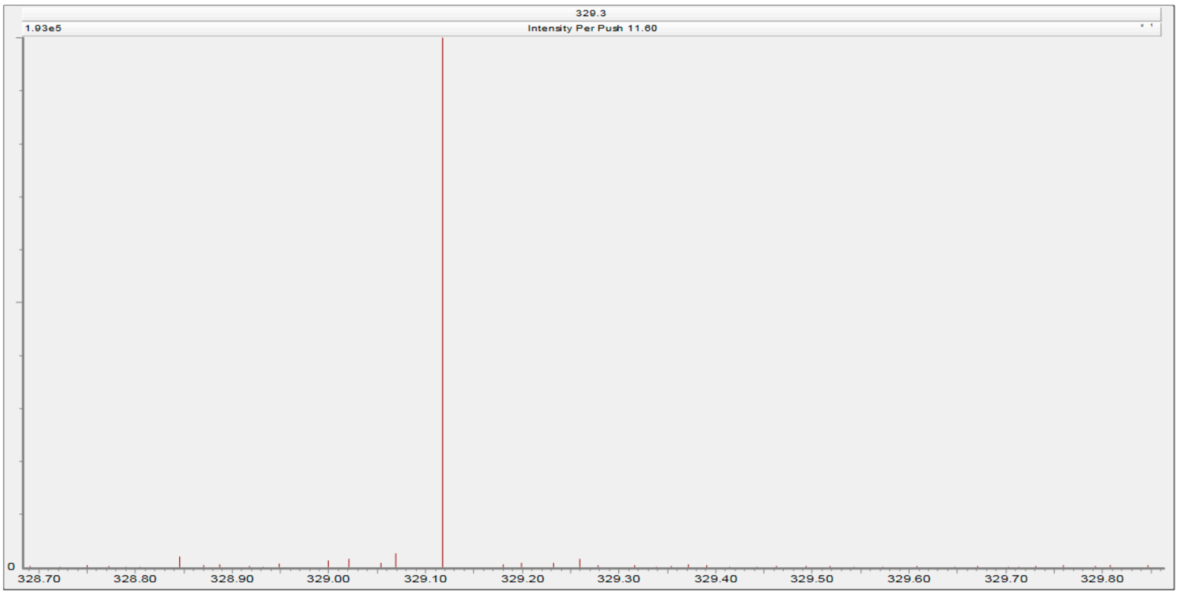

Figure S5. The AFB1 fragment ion with *m*/*z* 329.

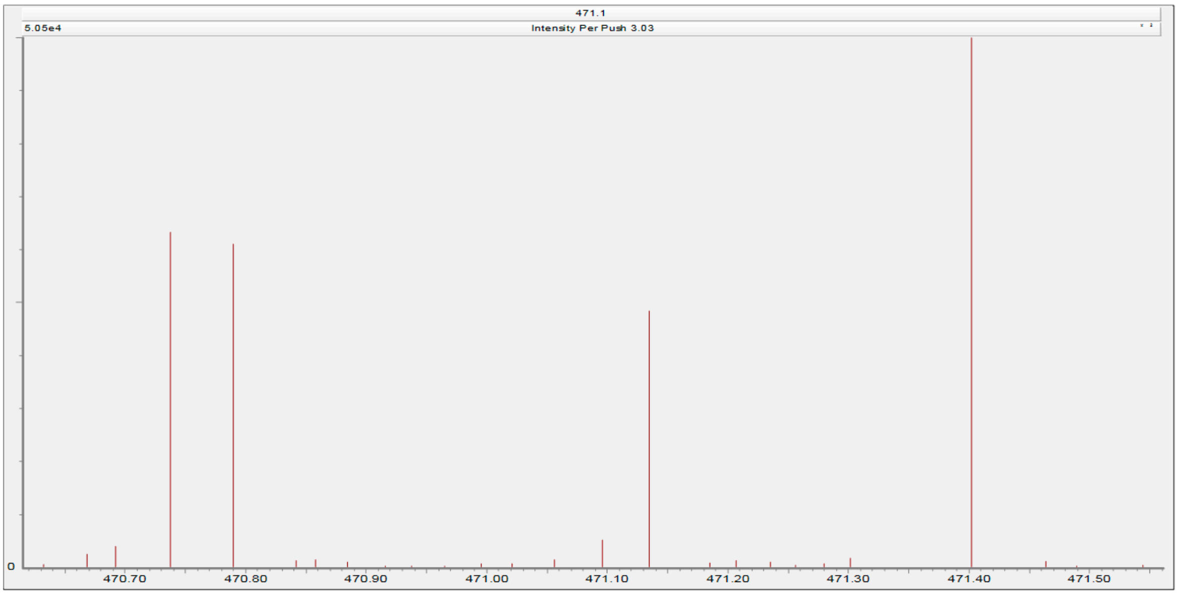

Figure S6. The fragment ion with *m*/*z* 471.

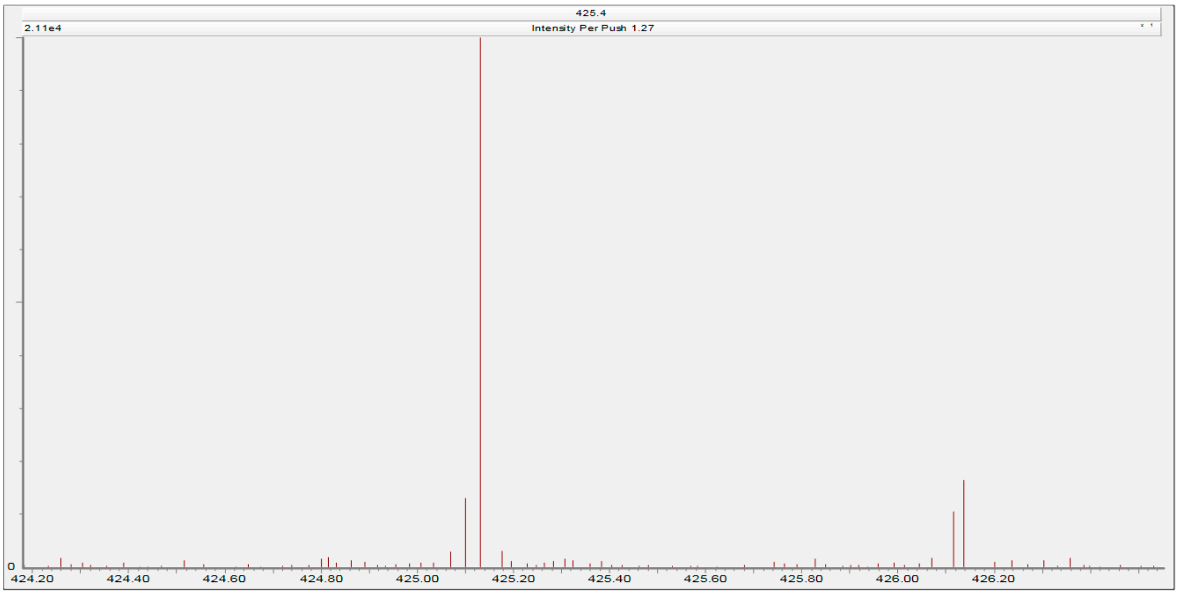

Figure S7. The fragment ion with *m*/*z* 425.

References

1. Sreerama, N.; Woody, R.W. Estimation of protein secondary structure from circular dichroism spectra: Comparison of contin, selcon, and cdsstr methods with an expanded reference set. *Anal. Biochem.* **2000**, *287*, 252–260.
